# Supplementary material for: Splice-Junction-Based Mapping of Alternative Isoforms in the Human Proteome
Source: Cell Rep. Author manuscript; Available in PMC 2020 Jan 15. (PMC6961840; doi:10.1016/j.celrep.2019.11.026)
Supplement: 3 [file NIHMS1546469-supplement-3.zip › DF2/PXD006675/VentricularSeptum-67-Q8TC07-SLSQSFENLLDEPAYGLIQK.pdf]

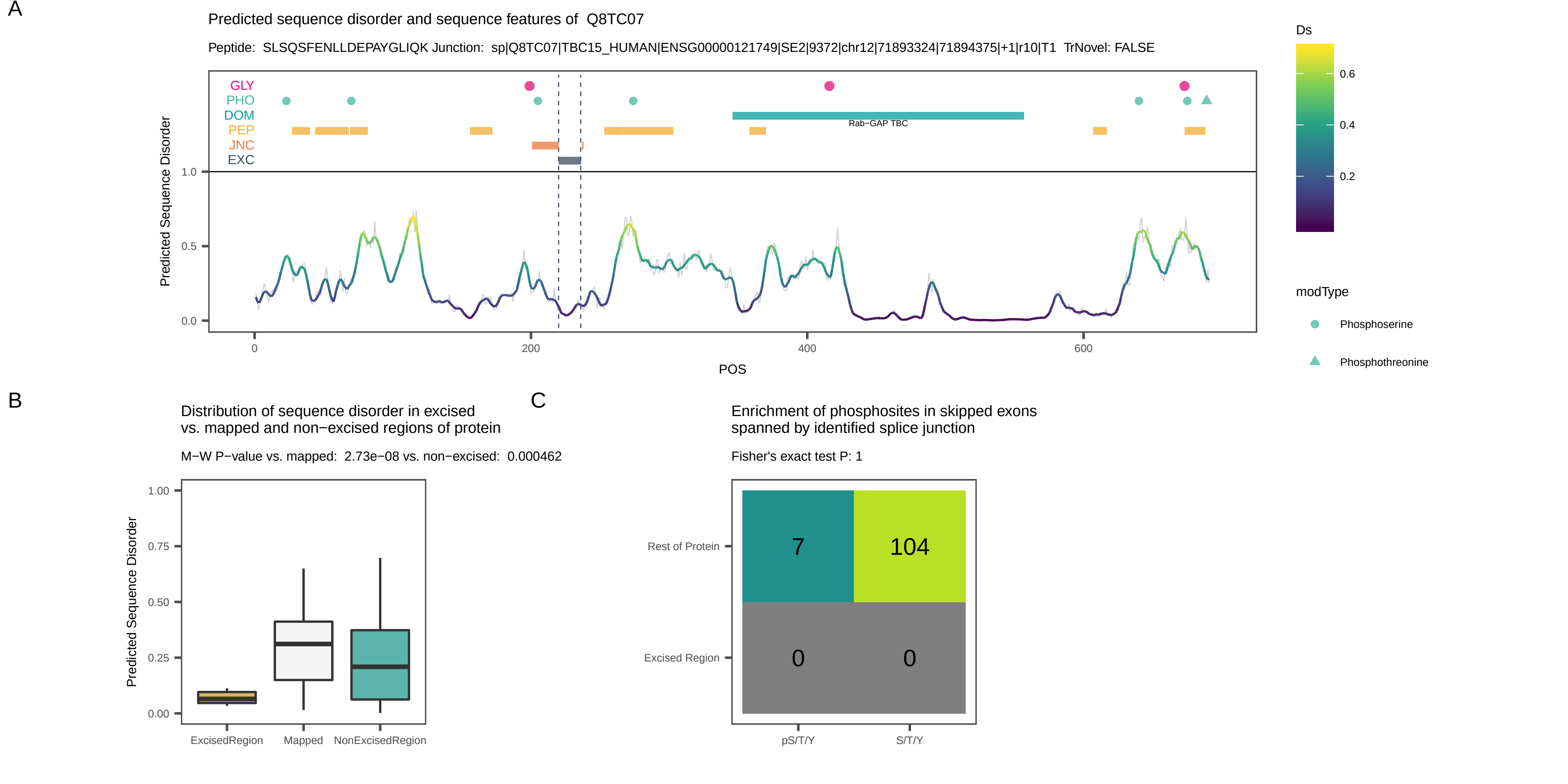
A
Predicted sequence disorder and sequence features of Q8TC07
Peptide: SLSQSFENLLDEPAYGLIQK Junction: sp|Q8TC07|TBC15_HUMAN|ENSG00000121749|SE2|9372|chr12|71893324|71894375|+1|r10|T1 TrNovel: FALSE
GLY
PHO
DOM
PEP
JNC
EXC
Rab−GAP TBC
Predicted Sequence Disorder
1.0
0.5
0.0
0
200
400
600
POS
Ds
0.6
0.4
0.2
modType
Phosphoserine
Phosphothreonine
B
Distribution of sequence disorder in excised vs. mapped and non−excised regions of protein
M−W P−value vs. mapped: 2.73e−08 vs. non−excised: 0.000462
Predicted Sequence Disorder
1.00
0.75
0.50
0.25
0.00
ExcisedRegion
Mapped
NonExcisedRegion
C
Enrichment of phosphosites in skipped exons spanned by identified splice junction
Fisher's exact test P: 1
Rest of Protein
Excised Region
7
104
0
0
pS/T/Y
S/T/Y
